# Susceptibility and prognosis of COVID-19 patients with cardiovascular disease

**DOI:** 10.1136/openhrt-2020-001310

**Published:** 2020-06-25

**Authors:** Brian Xiangzhi Wang

**Affiliations:** Imperial College London Faculty of Medicine, London, UK

**Keywords:** heart failure, hypertension, myocarditis

## Introduction

The outbreak of^5^ COVID-19, first identified in Wuhan, the capital of Hubei, China, in December 2019 has spread globally. Currently, we have no vaccine or specific antiviral treatment for the disease. Relatively little is known about the interplay between the disease with other comorbidities, including cardiovascular disease (CVD) and medications.^1^ Here, we outline what is known about the susceptibility of patients with CVDs to COVID-19 and the relative risk of these patients succumbing to severe illness.

## Disease transmission

COVID-19 is caused by the severe acute respiratory syndrome coronavirus 2 (SARS-CoV-2), phylogenetically related to the beta coronavirus SARS that caused a global outbreak in 2002. Coronaviruses contain four structural proteins, including spike (S), envelope, membrane and nucleocapsid.^2^ The S protein plays the most vital role in viral attachment, by binding to angiotensin-converting enzyme 2 (ACE2) in the lungs.^3^ In this way, these coronaviruses anchor to the lower respiratory tract of humans. The receptor binding domain of the SARS-CoV-2 S protein is therefore a likely target for the development of virus attachment inhibitors, neutralising antibodies and vaccines.

## Comorbidities in COVID-19 patients

Early studies in China investigated the prevalence of comorbidities in patients with COVID-19. In a study conducted on 138 hospitalised patients with COVID-19 at Zhongnan Hospital of Wuhan University from 1^st^ January 2020 to 28^th^ January 2020, half of these patients had at least one comorbidity, with hypertension (31%) and CVD (14.5%) being the two most common, and diabetes (10%) the third most common, demonstrating that CVD is common in patients with COVID-19.^4^ The criteria for inclusion as CVD for this study was not described. Patients with heart failure have previously been shown to be more likely to contract viral illnesses, although the mechanism underlying their increased risk has not been delineated. It may be, at least in part, due to the elevated levels of inflammatory mediators. The long-term increase in immune activity due to CVD, together with the reduced reserve work capacity, blunts the ability of these patients to clear low levels of the virus.

## Prognosis of COVID-19 patients with CVD

In a wider study that extracted data up to 29^th^ January 2020 from 552 hospitals in 30 provinces in China, they investigated the outcomes of 1099 patients.^5^ They investigated the incidence of severe illness using three primary end points—intensive care admission, use of a mechanical ventilator and death. The study identified that 5% of patients with COVID-19 were admitted to an intensive care unit, 2.3% of patients required a mechanical ventilator and 1.4% died (61% of mechanically-ventilated patients). Of the 261 patients with coexisting disorders, these primary end points were identified in 15% (39 patients). Hypertension was diagnosed in 165 patients, of which 24 patients had the primary end points (14.5%). Strikingly, of the 27 patients with coronary heart disease, 6 patients (22%) were positive for the primary end points.

A much larger study identified that among 44 672 patients that were confirmed to have COVID-19 as of 11^th^ February 2020, 2683 patients (12.8%) had hypertension and 873 (4.2%) had CVD. Importantly, this study also supported the notion that patients with CVDs were more likely to suffer severe illnesses from COVID-19 infection—10.5% of patients with CVDs and 6.0% with hypertension died, compared with the 0.9% in patients with no comorbidities.^6^ This provides significant evidence that CVD greatly increases the susceptibility of patients to severe illness.

## Cardiovascular complications of COVID-19 infection

Although the values vary between studies, they all indicate that individuals with underlying CVDs are more susceptible to COVID-19 and that these patients are more prone to critical conditions and death. It is well known that acute pulmonary infection, particularly with viral pathogens, can destabilise cardiac disease. Any deterioration of these diseases would, in turn, complicate the management of COVID-19. A local infection to the viral antigens can propagate into a systemic response. In patients with pneumonia, the most frequent causes of death are respiratory failure, sepsis and cardiac arrhythmias.^7^ The study presented by Wang and colleagues showed that of the 138 patients, while 27 (19.6%) had acute respiratory distress syndrome, there was acute cardiac injury in 10 (7.2%) patients.^4^ Although this was stated as being defined as troponin I elevation or new abnormalities on imaging, they did not provide clear classification of echocardiography parameters.

Viral infection can also lead to heart rhythm abnormalities. The study by Wang and colleagues identified that arrhythmia was reported in 23 (16.7%) patients with COVID-19.^4^ Similar to the echocardiography findings, arrhythmia classification parameters were not clearly outlined by the study. However, if COVID-19 does follow the trends of other beta coronaviruses, then arrhythmias are likely to be a common dysfunction in patients—Yu *et al* reported that in patients with SARS, tachycardia was present in 71.9% and bradycardia occurred in 14.9% as a transient event.^8^

To date, there is limited published data on the features of the myocardium in patients infected with SARS-CoV-2. Future studies must identify, via biopsies, the histological features of the myocardium. Alternatively, cardiac MRI could provide crucial non-invasive insight into the nature of the cardiac injury in these patients.

## Protection from infection

One clinical observation that may be key in understanding susceptibility to COVID-19 is that while severe infection is frequently identified in the elderly population, especially in those with chronic comorbidities, infection is relatively infrequent in children. It has been postulated that cross-protective antibodies from multiple upper respiratory tract infections caused by the common cold-causing alpha coronaviruses enable children to mount an immune response that enables clearance of the virus. Alternatively, as ACE2 expression in the lower respiratory tract increases during ageing, children may have a lower expression of the entry receptor for SARS-CoV-2 entry.^9^

## CVD medication

Medications that modulate the renin-angiotensin pathway could play a significant role in the transmission of SARS-CoV-2. Animal studies have demonstrated that intravenous infusion of ACE inhibitors (ACEIs) and angiotensin II receptor blockers (ARBs) increase the prevalence of ACE2 in the cardiopulmonary circulation.^10^ This led to growing concerns that medications acting on the renin-angiotensin-aldosterone system increased the risk of patients contracting COVID-19 or having a more severe illness. Reynolds and colleagues identified that among 12 954 patients in the New York University electronic health records who had tests results, there were no significant association between any of the five common classes of antihypertensive medications recorded and the likelihood of testing positive for COVID-19 or requiring intensive care unit admission, the use of mechanical ventilation or death.^11^ A population-based study by Mancia and colleagues on patients in Lombardy, Italy, identified that ACEIs and ARBs were more frequent in patients with COVID-19 than controls, attributed to the higher prevalence of cardiovascular disease.^12^ Mehra and colleagues, analysing a database from 169 hospitals, also identified an increased risk of severe illness in patients with CVD but could not attribute outcomes to the use of ACEIs or ARBs, despite ACEIs being more commonly used in those that survived the illness.^13^ Key limitations to all these studies are the lack of robust diagnostic tests and lack of randomisation, which opens the opportunity for confounding factors.

## Future considerations

In recent weeks, numerous studies have investigated potential relationships between medications and COVID-19 disease progression. However, to date, there is still no consensus and all studies have demonstrated potential confounding factors in interpreting the data. Random clinical trials evaluating the role of medication that act on the renin-angiotensin-aldosterone pathway are needed before any conclusions can be reached regarding a potential relationship between these agents and the progression of COVID-19.

## Conclusion

COVID-19 has emerged as an acute infectious disease. However, as it may become a chronic epidemic similar to influenza, even if infections do become controlled, we must be prepared for re-emergence of COVID-19 as well as future outbreaks with other coronaviruses. We must understand the association that ACEIs and ARBs have susceptibility to COVID-19 as well as the prognosis following infection. This could help us delineate the important mechanisms for disease transmission and fight the spread of the disease.
